# Ultrasound Radiomics Effective for Preoperative Identification of True and Pseudo Gallbladder Polyps Based on Spatial and Morphological Features

**DOI:** 10.3389/fonc.2020.01719

**Published:** 2020-09-11

**Authors:** Hai-xia Yuan, Qi-hui Yu, Yan-qun Zhang, Qing Yu, Qi Zhang, Wen-ping Wang

**Affiliations:** ^1^Department of Ultrasound, Zhongshan Hospital of Fudan University, Shanghai, China; ^2^Department of Ultrasound, Xiamen Branch, Zhongshan Hospital of Fudan University, Xiamen, China; ^3^The SMART (Smart Medicine and AI-based Radiology Technology) Lab, School of Communication and Information Engineering, Shanghai University, Shanghai, China; ^4^Shanghai Institute of Medical Imaging, Shanghai, China; ^5^Hangzhou YITU Healthcare Technology, Hangzhou, China

**Keywords:** gallbladder true-polyps, gallbladder pseudo-polyps, ultrasound radiomics, gallbladder cholesterol polyp, gallbladder adenoma, preoperative identification

## Abstract

**Purpose:** To explore the value of ultrasound radiomics in the preoperative identification of true and pseudo gallbladder polyps and to evaluate the associated diagnostic accuracy.

**Methods:** Totally, 99 pathologically proven gallbladder polyps in 96 patients were enrolled, including 58 cholesterol polyps (55 patients) and 41 gallbladder tubular adenomas (41 patients). Features on preoperative ultrasound images, including spatial and morphological features, were acquired for each lesion. Following this, two-stage feature selection was adopted using Fisher's inter-intraclass variance ratios and Z-scores for the selection of intrinsic features important for differential diagnosis achievement with support vector machine use.

**Results:** Eighty radiomic features were extracted from each polyp. Eight intrinsic features were identified after two-stage selection. The contrast 14 (Cont14) and entropy 6 (Entr6) values in the cholesterol polyp group were significantly higher than those in the gallbladder adenoma group (4.063 ± 1.682 vs. 2.715 ± 1.867, *p* < 0.001 for Cont14; 4.712 ± 0.427 vs. 4.380 ± 0.720, *p* = 0.003 for Entr6); however, the homogeneity 13 (Homo13) and energy 8 (Ener8) values in the cholesterol polyp group were significantly lower (0.500 ± 0.069 vs. 0.572 ± 0.057, *p* < 0.001 for Homo13; 0.050 ± 0.023 vs. 0.068 ± 0.038, *p* = 0.002 for Ener8). These results indicate that the pixel distribution of cholesterol polyps was more uneven than that of gallbladder tubular adenomas. The dispersion degree was also significantly lower in the cholesterol polyp group than the gallbladder adenoma group (0.579 ± 0.054 vs. 0.608 ± 0.041, *p* = 0.005), indicating a lower dispersion of high-intensity areas in the cholesterol polyps. The long axis length of the fitting ellipse (Maj.Len), diameter of a circle equal to the lesion area (Eq.Dia) and perimeter (Per) values in the cholesterol polyp group were significantly lower than those in the gallbladder adenoma group (0.971 ± 0.485 vs. 1.738 ± 0.912, *p* < 0.001 for Maj.Len; 0.818 ± 0.393 vs. 1.438 ± 0.650, *p* < 0.001 for Eq.Dia; 2.637 ± 1.281 vs. 5.033 ± 2.353, *p* < 0.001 for Per), demonstrating that the cholesterol polyps were smaller and more regular in terms of morphology. The classification accuracy, sensitivity, specificity, and area under the curve values were 0.875, 0.885, 0.857, and 0.898, respectively.

**Conclusions:** Ultrasound radiomic analysis based on the spatial and morphological features extracted from ultrasound images effectively contributed to the preoperative diagnosis of true and pseudo gallbladder polyps and may be valuable in their clinical management.

## Introduction

With the development of high-resolution ultrasound equipment and increased frequency of periodic health examinations, numerous gallbladder polyps are now diagnosed at an early phase. Although the reported incidence rate in adults is~0.3–12.3%, only about 5% of polyps are true polyps (1, 2). Postoperative pathological gallbladder polyp types include cholesterol polyps, inflammatory polyps, adenomyomas, adenomas, and early gallbladder cancer. Gallbladder cholesterol polyps and gallbladder adenoma polyps are the two most commonly observed types and are associated with different clinical procedures. Gallbladder cholesterol polyps are a type of pseudo polyps and are usually caused by the accumulation of cholesterol crystals in the inner wall of the gallbladder that are swallowed by macrophages. This subsequently promotes the formation of foam cells at the surface of the gallbladder mucosa; most cholesterol polyps tend to remain in a benign state (3, 4). Inversely, gallbladder adenomas are true polyps and usually coexist with atypical hyperplasia; they tend to progress to gallbladder cancer (5, 6). Therefore, the preoperative identification of gallbladder true polyps is vital.

At present, the accurate identification of the aforementioned polyps before cholecystomy using the existing imaging techniques is extremely challenging. Ultrasonography is the preferred imaging method owing to its characteristics that include radiation absence, clear imaging and scanning section flexibility. However, few studies have focused specifically on how gallbladder cholesterol polyps and adenomas can be distinguished from each other. Park et al. (7) found different types of gallbladder adenomas and cholesterol polyps in progression by the application of endoscopic ultrasound, the use of which is limited in clinical practice due to its invasiveness. With the use of contrast-enhanced ultrasound, our previous study (8) revealed that gallbladder adenomas exhibit uniformly eccentric enhanced characteristics and slower regression compared to gallbladder cancer. However, as some hospitals do not use contrast-enhanced ultrasound, the distinction of gallbladder adenomas from cholesterol polys is a tremendous challenge for radiologists. Further reliable and objective methods are needed for a larger number of imaging features to be obtained for differential diagnosis.

Nowadays, surgical guidelines recommend that gallbladder polyps of size >1 cm be surgically resected as gallbladder adenomas and carcinomas are larger than benign polyps (9). However, of 1,541 cases of gallbladder polyps investigated in our hospital from January 2011 to November 2018, only ~30% of gallbladder polyps were pathologically proven as being gallbladder adenomas, adenomas with severe atypia, or cancerous adenomas, indicating that the remaining 70% were pseudo gallbladder polyps, including cholesterol polyps, adenoma-like hyperplasia, and inflammatory polyps. Therefore, there is an urgent need for clinical surgery aimed at the identification of a novel imaging method with higher diagnostic accuracy that may allow for the avoidance of unnecessary cholecystectomy, reduce the wastage of medical resources, and relieve patient suffering.

The field of radiomic technology based on artificial intelligence (AI) has been developing rapidly in recent years, with computers processing massive datasets through layered mathematical models that can detect patterns not otherwise decipherable using biostatistics (10). Many researchers have made progress in the field of radiomics. Wang et al. (11) showed that the newly developed deep learning radiomics of elastography (DLRE) was valuable in liver fibrosis stage prediction. Liu et al. (12) developed a radiomics model that incorporated radiomics signatures and independent clinicopathological risk factors, that allowed for the performance of the individualized, non-invasive prediction of pathologic complete response to neoadjuvant chemoradiotherapy in patients with locally advanced rectal cancer. Song et al. (13) demonstrated the individualized prediction of progression-free survival probability associated with epidermal growth factor receptor tyrosine kinase inhibitor therapy in non-small cell lung cancer on the basis of computed tomography features. In general, AI is widely used in the field of medical radiomics analysis, with computers capturing changes in the protein genes on macroscopic images using information of a higher dimension. This is expected to provide accurate and reliable diagnostic recommendations for doctors' clinical decisions (14–17).

In this study, we aimed to retrospectively analyse the preoperative two-dimensional ultrasound images of patients with gallbladder adenomas and gallbladder cholesterol polyps. Multiple groups of imaging features were extracted automatically for the detection of early imaging differences between the two diseases so as to provide accurate diagnosis.

## Materials and Methods

### Patients

Approval was obtained from the Institutional Ethics Committee for the retrospective review of images and patients' medical records(Y2020-188).

The exclusion criteria were as follows: (1) diagnosis of gallbladder carcinoma on previous imaging; (2) insufficient liver, kidney or heart function; (3) presence of a thickened gallbladder wall lesion; and (4) imaging scanning demonstrated liver metastasis.

**Figure 1:**
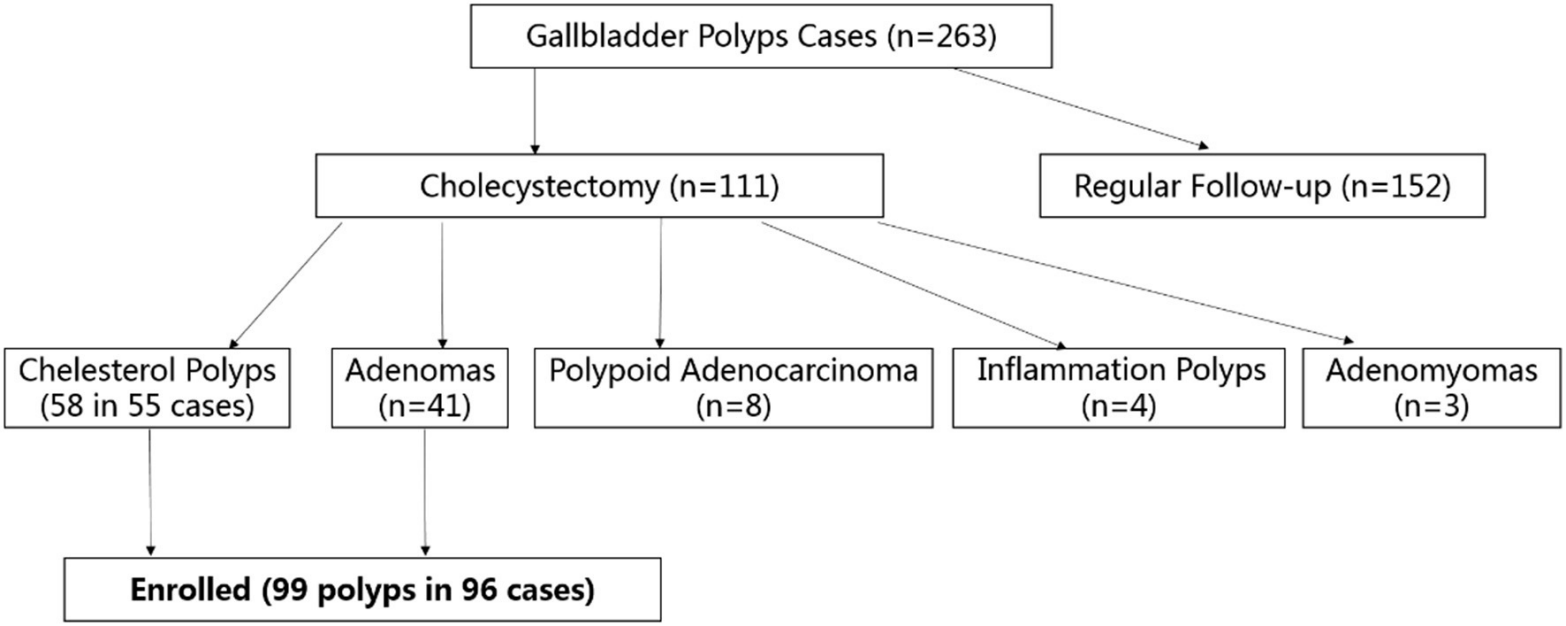
Flow chart of participant enrolment. Totally, 263 patients with gallbladder polyps (size >7 mm) were referred to our hospital: 152 cases chose clinical follow-up, 111 underwent cholecystectomy for pathological proven cholesterol polyps (*n* = 58), gallbladder tubular adenomas (*n* = 41), gallbladder polypoid adenocarcinomas (*n* = 8), inflammation polyps (*n* = 4), and adenomyomas (*n* = 3). Finally, 99 gallbladder polyps (cholesterol polyps and adenomas) in 96 patients were enrolled.

From July 2018 to December 2019, 263 patients with gallbladder polyps (size >7 mm) were referred to our hospital for surgical treatment (Figure 1), and all of them underwent ultrasound. After a discussion with their surgeons, 152 cases chose clinical follow-up, and 111 underwent cholecystectomy. The polyps were pathologically proven as being cholesterol polyps (*n* = 58) in 55 patients and gallbladder tubular adenomas (*n* = 41) in 41 patients. Gallbladder polypoid adenocarcinomas (*n* = 8), inflammation polyps (*n* = 4), and adenomyomas (*n* = 3) were also observed. Patients with polypoid adenocarcinomas, inflammation polyps and adenomyomas were excluded from this study owing to the small sample size. Finally, we enrolled 99 gallbladder polyps (cholesterol polyps and adenomas) in 96 patients (40 men and 56 women; mean age 36.5 years, age range 27–71 years).

### Ultrasound Scanning and Instruments

All patients fasted for at least 8 h before undergoing ultrasound examination. Gray-scale and color Doppler ultrasound were performed. The target area was magnified to ensure the ideal plane for the display of the whole gallbladder and adjacent liver parenchyma. Ultrasound was performed by two experienced technologists using one of the following ultrasonographic systems: Aplio 500 (Canon Healthcare, Japan; PVT-375BT, 1.9–6 MHz), Ascendus (Hitachi Medical Systems, Japan; EUP-C715, 1–5 MHz), Resona 7s (Mindray Medical Systems, China; SC5-1U, 1–5 MHz), and Mylab Twice (Esaote Medical Systems, Italy, CA431, 1–5 MHz). The maximum diameter of the polyp was measured, and the original ultrasound images of the lesion were captured for further analysis.

### Ultrasound Radiomics Analysis Procedure

#### Overall Design

**Figure 2:**
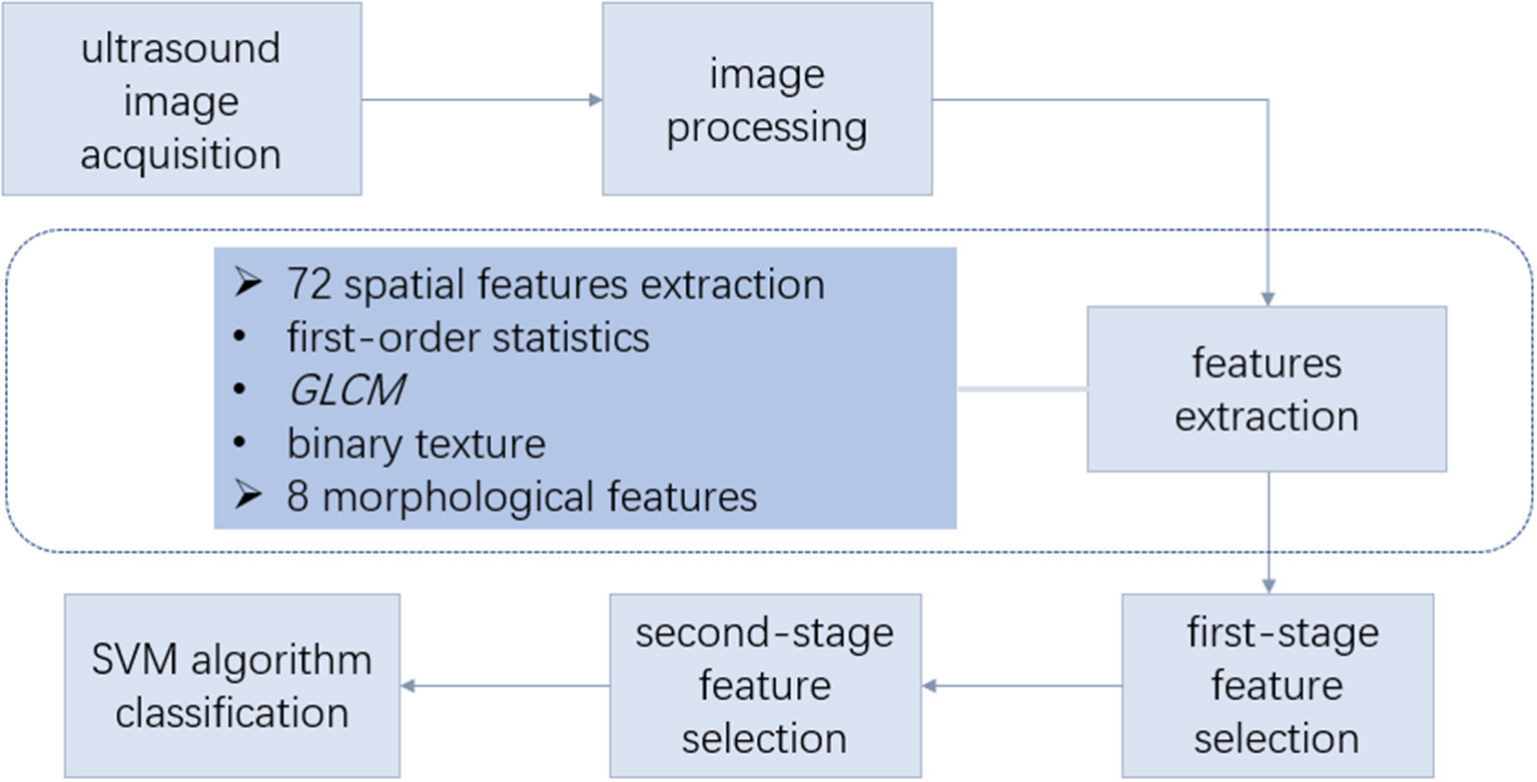
Overall design of radiomic analysis based on ultrasound images. The radiomic analysis comprised the following steps: (1) acquisition of the ultrasound images and lesion contour delineation; (2) image processing to obtain the mask image; (3) spatial feature extraction based on the ultrasonic gray-scale and binary image; (4) morphological feature extraction; (5) first-stage feature selection; (6) second-stage feature selection; (7) support vector machine (SVM) classification using the selected features. GLCM, gray-level co-occurrence matrix.

The radiomic analysis based on ultrasound images comprised seven steps, as shown in Figure 2.

#### Image Processing

**Figure 3:**
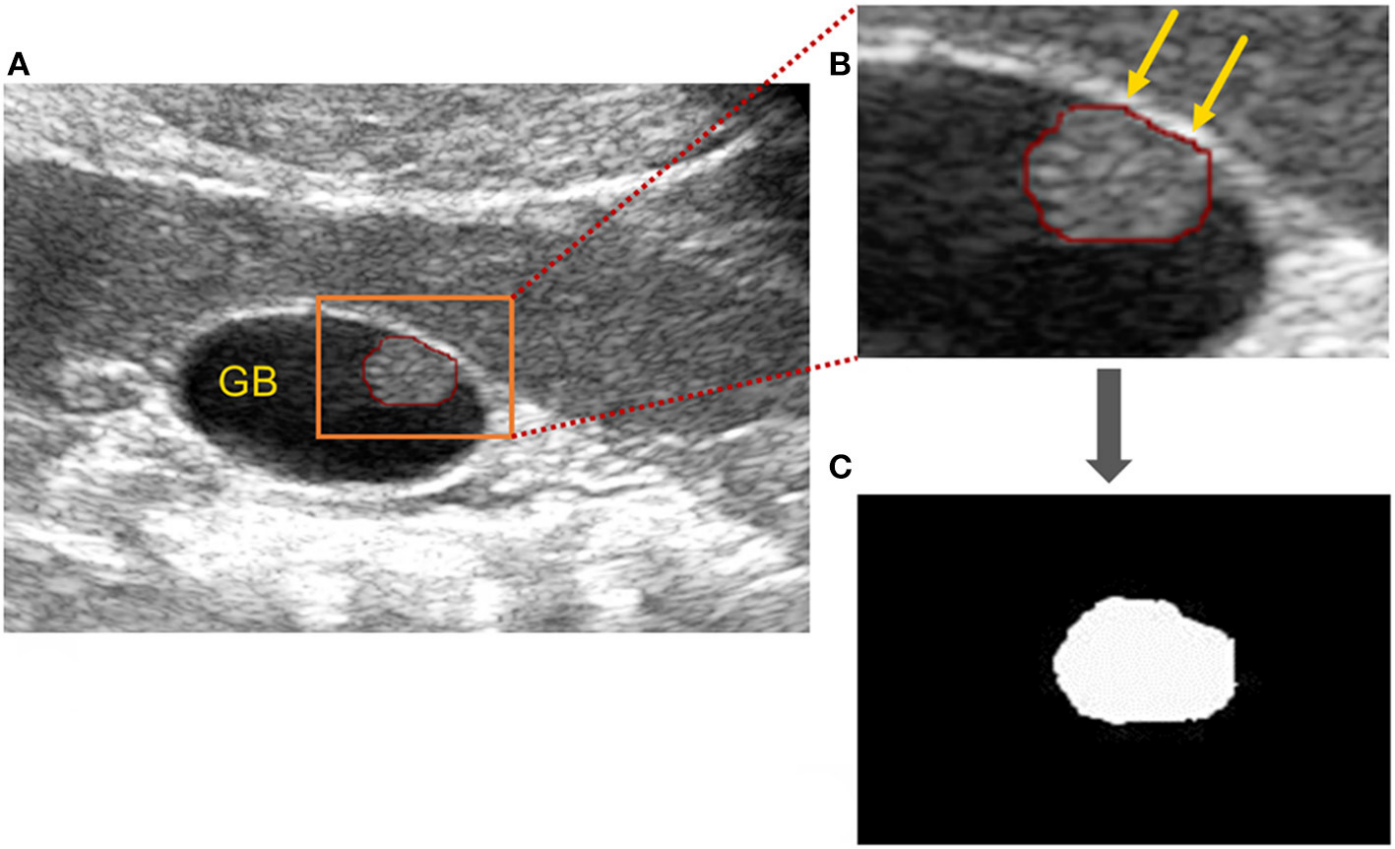
Image processing of gallbladder cholesterol polyps. **(A)** Original ultrasound image of a gallbladder polyp: the polyp is circled (red line) and the area (orange rectangle) showing the gallbladder polyp) has been zoomed in partially to **(B)**. **(B)** Partially enlarged gallbladder polyp (orange arrows). **(C)** Mask of gallbladder polyp after binarisation processing.

In this retrospective study, the ultrasound images of cholesterol polyps and gallbladder adenomas were acquired, and the edge of each lesion was circled with a red curve by the drawing software (Figure 3A). Then, the images were binarised with the thresholding method to obtain mask images on which the outline was filled with white inside and the rest set to black. The area (orange rectangle) showing the gallbladder polyp was zoomed partially (Figure 3B), and the mask of the gallbladder polyp lesion was shown as in Figure 3C.

#### Spatial Feature Extraction

The imaging features of the lesion were extracted based on the original ultrasound image and the corresponding mask image. Some spatial features were extracted based on the ultrasonic gray-scale image, which included first-order statistic features and gray-level co-occurrence matrix (GLCM) texture features. Additionally, binary texture spatial features were extracted based on the ultrasonic binary mask image for the reflection of pixel distribution inside the lesion.

The first-order statistic features included the mean (IMean), median (IMedian), standard deviation, coefficient of variation, histogram entropy, skewness, and kurtosis of the pixels within the lesion. The corresponding ratio of the median (mean) of the pixels was calculated, which was within the lesion and within the reference area (the rectangular area expanding outwards from the lesion), and the ratio was defined as RImedian (RImean).

The GLCM is an important technique for texture analysis (18), which represents the characteristics of the intensity distribution and respective distance of the intensity levels in the original image. In this study, the GLCM texture features were of four types: energy (Ener), contrast (Cont), entropy (Entr), and homogeneity (Homo), and each type of GLCM feature was constructed for different values of offset d. Here, d was an integer between 1 and 15 pixels. Therefore, each type of GLCM feature included 15 texture features; a total of 60 GLCM texture features was extracted for each lesion.

The binary texture features included the following: the area ratio (AR), which denotes the ratio of the high-intensity area to the whole lesion area; center deviation degree, which characterizes the normalized distance between each pixel point in the high-intensity area of the lesion and the center point of the lesion; and dispersion degree (DD), which characterizes the mean of the normalized Euclidean distance between each pixel point in the high-intensity area of the lesion and the center point of the high-intensity area (19).

#### Morphological Feature Extraction

**Figure 4:**
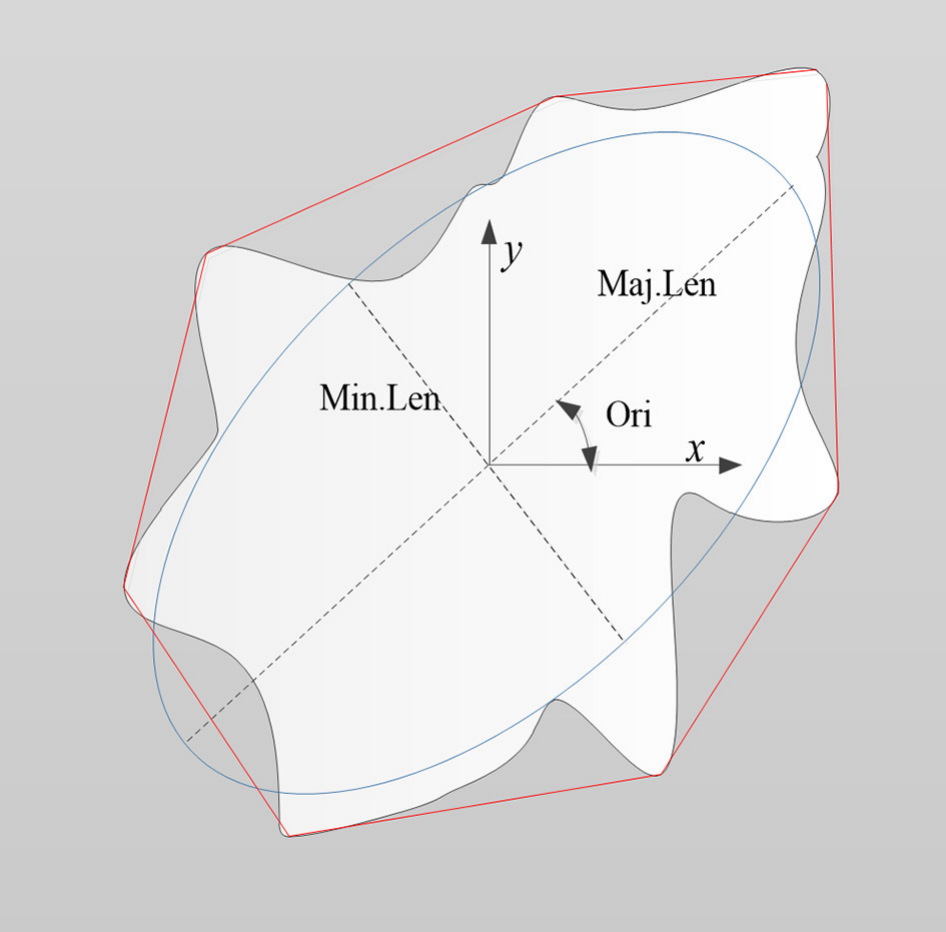
Schematic diagram illustrating the morphological features of a lesion (denoted as the white region). Features included the area of the lesion (Area), area of minimum convex polygon encompassing the lesion (C.area; denoted as the area inside the red line), long axis length (Maj.Len), and short axis length (Min.Len) of the fitting ellipse (blue line), perimeter (Per), orientation (Ori), equivalent diameter (Eq.Dia), and solidity (Sol).

As shown in Figure 4, the morphological features of the lesion were extracted, including the area of the lesion (Area), area of the minimum convex polygon corresponding to the lesion (C.area), long axis length (Maj.Len), and short axis length (Min.Len) of the fitting ellipse with the same standard second order center distance as the lesion, number of contour pixel points of the lesion (perimeter, Per), angle between the long axis of the fitting ellipse and X-axis (orientation, Ori), diameter of a circle equal to the lesion area (equivalent diameter, Eq.Dia), and ratio of the lesion area to convex area (solidity, Sol).

#### First-Stage Feature Selection

Assuming the features were normally distributed, the non-paired *t*-test was used to analyse the features of the gallbladder cholesterol polyps and gallbladder tubular adenomas. Otherwise, the Kruskal–Wallis test was used to analyse the features. Here, *p*-values lower than 0.05 indicated statistical significance.

In order to further enhance the reliability of the features and select intrinsic features from among all the significant features, we adopted two indicators—*Fv* and *F* values. Here, the *Fv*-value was Fisher's inter-intraclass variance ratio and *F*-value was defined using Z-scores (20):

(1) $$\begin{array}{l} F_{\text{v}}=\frac{\left|{\bar{x}}_{0}-{\bar{x}}_{1}\right|}{\sqrt{\left(\sigma_{0}^{2}+\sigma_{1}^{2}\right)}} \end{array}$$

(2) $$\begin{array}{l} f=|mean_{i}\left(z\text{-}score_{oi}\right)-mean_{i}\left(z\text{-}score_{1i}\right)|\\ \begin{array}{l} \;=|mean_{i}\left(\frac{x_{0i}-{\bar{x}}_{0}}{\sigma_{0}}\right)-mean_{i}\left(\frac{x_{1i}-{\bar{x}}_{1}}{\sigma_{1}}\right)| \end{array} \end{array}$$

where the subscripts 0 and 1 represented the gallbladder cholesterol polyps and gallbladder tubular adenomas, respectively, $\bar{x}$ and σ denoted the mean and standard deviation of a feature. *X*_0_*i* represented the *i*-th data of a feature in class 0, and *X*_1_*i* represented the *i*-th data of a feature in class 1.

Considering the presence of a large number of GLCM features (60) and the likelihood of internal redundancy, we first retained the feature with the largest *Fv* value in each type of GLCM feature as the representative feature of GLCM. In addition to the GLCM features, other statistically significant features were retained.

#### Second-Stage Feature Selection

Next, we selected a few more important features after first-stage feature selection. In terms of spatial features, we retained the features that satisfied both the following criteria: (1) *Fv* value was greater than the median *Fv* value of the alternative spatial domain features. (2) F value was greater than the median corresponding F value of the alternative spatial domain features. Similarly, in terms of morphological features, we retained the features that satisfied both the following criteria: (1) *Fv* value was greater than the median *Fv* value of the alternative morphological features. (2) F value was greater than the median corresponding F value of the alternative morphological features.

#### Classification

For the classification of features, we used the supervised support vector machine (SVM) algorithm. The SVM is used for the identification of a decision boundary to maximize the margin between two classes and is a very popular classification method (21). First, we divided the data set into the training set and test set in a ratio of 6:4. In the training set, we used 5-fold cross validation for the identification of the optimal model of the features, which was then used for the test set classification. Finally, we acquired the classification performance of the test set, including the classification accuracy, classification sensitivity, specificity, Youden index, and area under the curve (AUC).

## Results

### Features After First-Stage Feature Selection

The enrolled cases were confirmed by surgical pathology, and included 58 cases of cholesterol polyps in 55 patients and 41 cases of gallbladder tubular adenomas in 41 patients. Each case corresponded to 72 spatial features and eight morphological features. Finally, 69 significant features were obtained from among all the features of the two diseases, including 52 GLCM features. The spatial and morphological features obtained after first-stage feature selection are shown in Table 1, in which, if a feature was normally distributed, its mean and standard deviation are shown, otherwise its median and interquartile range are given.

**Table 1:** Features showing statistical significance.

	**Features**	**Gallbladder cholesterol polyps (Class 0)**	**Gallbladder tubular adenomas (Class 1)**	***P***	***F*v**	***F***
Spatial features	CDD	0.614 ± 0.052	0.639 ± 0.031	0.007	0.413	0.545
	DD	0.579 ± 0.054	0.608 ± 0.041	0.005	0.421	0.564
	AR	0.536 ± 0.119	0.580 ± 0.090	0.046	0.298	0.408
	Imedian	88.474 ± 24.727	104.256 ± 31.886	0.007	0.391	0.550
	Imean	88.237 ± 24.154	102.897 ± 30.604	0.009	0.376	0.530
	CoV	0.307 ± 0.105^*^	0.263 ± 0.084	0.001	0.322	0.674
	Kurtosis	2.858 ± 0.858^*^	3.279 ± 1.051^*^	0.012	0.310	0.235
	Cont14	4.063 ± 1.682^*^	2.715 ± 1.867	<0.001	0.536	0.883
	Ener8	0.050 ± 0.023^*^	0.068 ± 0.038^*^	0.002	0.414	0.583
	Homo13	0.500 ± 0.069	0.572 ± 0.057	<0.001	0.796	0.980
	Entr6	4.712 ± 0.427^*^	4.380 ± 0.720	0.003	0.396	0.564
Morphological features	Area	0.525 ± 0.489^*^	1.623 ± 1.405^*^	<0.001	0.738	0.883
	Maj.Len	0.971 ± 0.485^*^	1.738 ± 0.912^*^	<0.001	0.742	1.045
	Min.Len	0.651 ± 0.298^*^	1.135 ± 0.602^*^	<0.001	0.720	1.117
	C.Area	0.537 ± 0.511^*^	1.707 ± 1.423^*^	<0.001	0.774	0.882
	Eq.Dia	0.818 ± 0.393	1.438 ± 0.650^*^	<0.001	0.816	1.131
	Ori	15.261 ± 41.013^*^	33.606 ± 44.117	0.002	0.305	0.562
	Per	2.637 ± 1.281^*^	5.033 ± 2.353^*^	<0.001	0.894	1.124
	Sol	0.980 ± 0.030^*^	0.963 ± 0.053^*^	0.005	0.273	0.283

* *Parameters with non-normal distribution*.

*CDD, center deviation degree; DD, dispersion degree; CoV, coefficient of variance; C.area, area of minimum convex polygon; Maj.Len, long axis length of the fitting ellipse; Min.Len, short axis length of the fitting ellipse; Per, perimeter; Ori, orientation; Eq.Dia, diameter of a circle equal to the lesion area; Sol, solidity; AR, area ratio; IMean, mean of the pixels within the lesion; Imedian, median of the pixels within the lesion; Cont, Contrast; Ener, energy; Homo, homogeneity; Entr, entropy*.

### Features After Second-Stage Feature Selection

In the second-stage feature selection, we retained the spatial features that satisfied *Fv*> = 0.396 and *F*> = 0.564. Similarly, we retained the morphological features that satisfied *Fv*> = 0.740 and *F*> = 0.964. Finally, a total of eight features was selected, as shown in Table 2.

**Table 2:** Features filtered using the *Fv* value and *F* value.

	**Features**	**Gallbladder cholesterol polyps**	**Gallbladder tubular adenomas**	***P***	***F*v**	***F***
Spatial features	Cont14	4.063 ± 1.682^*^	2.715 ± 1.867	<0.001	0.536	0.883
	Ener8	0.050 ± 0.023^*^	0.068 ± 0.038^*^	0.002	0.414	0.583
	Homo13	0.500 ± 0.069	0.572 ± 0.057	<0.001	0.796	0.980
	Entr6	4.712 ± 0.427^*^	4.380 ± 0.720	0.003	0.396	0.564
	DD	0.579 ± 0.054	0.608 ± 0.041	0.005	0.421	0.564
Morphological features	Maj.Len	0.971 ± 0.485^*^	1.738 ± 0.912^*^	<0.001	0.742	1.045
	Eq.Dia	0.818 ± 0.393	1.438 ± 0.650^*^	<0.001	0.816	1.131
	Per	2.637 ± 1.281^*^	5.033 ± 2.353^*^	<0.001	0.894	1.124

*DD, dispersion degree; Maj.Len, long axis length of the fitting ellipse; Per, perimeter; Eq.Dia, diameter of a circle equal to the lesion area; Cont, Contrast; Ener, energy; Homo, homogeneity; Entr, entropy*.

As Table 2 indicates, in terms of spatial features, the Cont14 and Entr6 values in the cholesterol polyp group were significantly higher than those in the gallbladder adenoma group, but the Homo13 and Ener8 values in the cholesterol polyp group were significantly lower than those in the gallbladder adenoma group. These results indicate that the pixel distribution of the cholesterol polyp lesions was more uneven than that of the gallbladder tubular adenomas. The DD was also significantly lower in the cholesterol polyps than gallbladder adenomas, indicating a lower degree of dispersion of the highlight area in the cholesterol polyps. In addition, in terms of morphological characteristics, the Maj.Len, Eq.Dia and Per values in the cholesterol polyp group were significantly lower than those in the gallbladder adenoma group, demonstrating that the cholesterol polyps were smaller and more regular in appearance than the gallbladder tubular adenomas.

**Figure 5:**
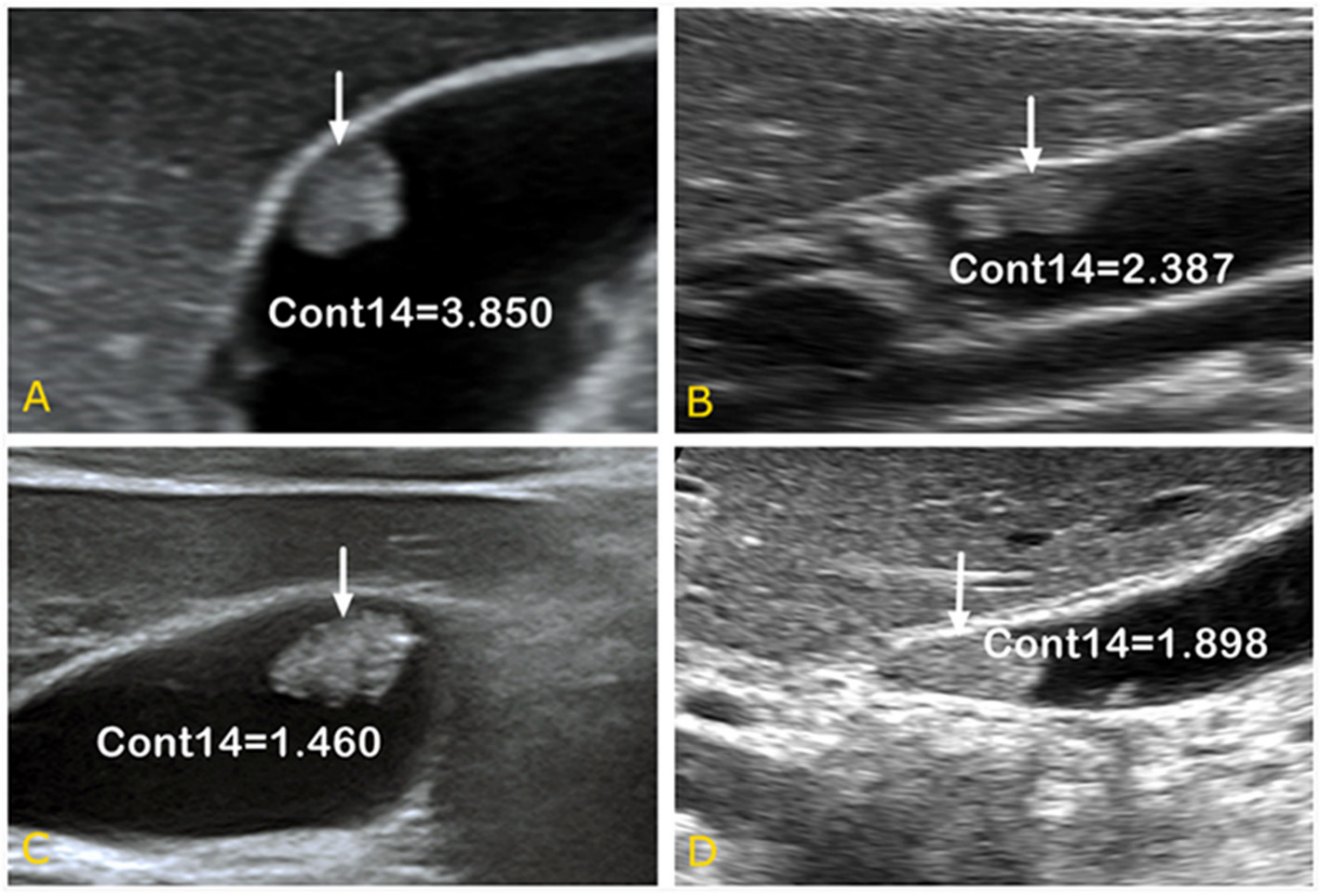
Ultrasound images of gallbladder cholesterol polyps **(A,B)** and gallbladder tubular adenomas **(C,D)**. When combined with spatial parameter analysis, the Cont14 values were significantly higher (3.850 and 2.387) than those of the gallbladder adenomas (1.460 and 1.898), indicating the pixel distribution of the cholesterol polyp lesions was more uneven than that of the gallbladder tubular adenomas. Cont, contrast.

As shown in Figure 5, it is very hard to manually and visually distinguish gallbladder adenomas (Figures 5C,D) from cholesterol polyps (Figures 5A,B) based on their ultrasound images. Using radiomic analysis, the Cont14 value was found to be significantly higher (3.850 and 2.387) than that of the gallbladder adenomas (1.460 and 1.898). These results indicate that the pixel distribution of the cholesterol polyp lesions was more uneven than that of the gallbladder tubular adenomas, and that ultrasound radiomics based on spatial and morphological features may be valuable for the differential diagnosis of these two diseases.

### Classification Results of SVM

**Figure 6:**
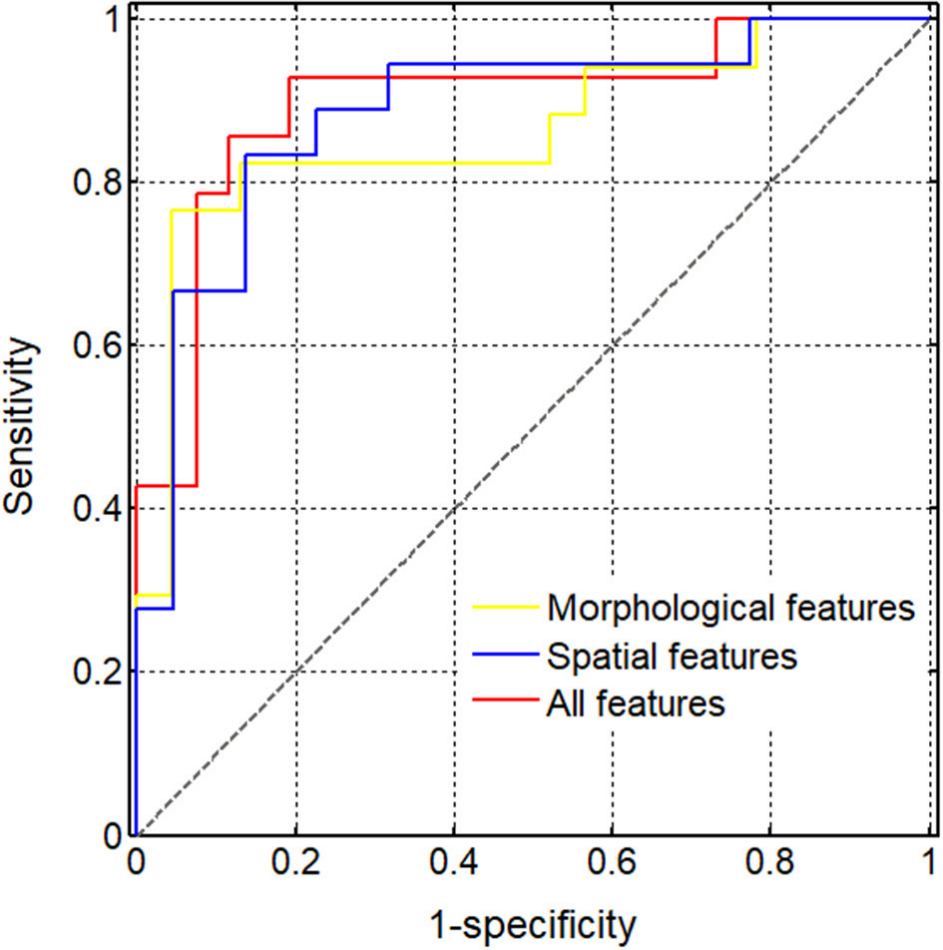
Diagnostic performance of different support vector machine (SVM) models. The (areas under the curve) AUCs obtained using the SVM models of various feature sets were compared, including three morphological features (0.862), five spatial features (0.886), and all eight selected features (0.898).

Finally, we used the SVM to obtain the optimal models of five spatial features and three morphological features. The classification performance in the test set (Table 3) indicated that the accuracy of the spatial feature model was higher than that of the morphological feature model, but the sensitivity and specificity the spatial feature model were more unbalanced than those of the morphological feature model. When we applied the SVM to all eight features for the classification performance of the test set, the accuracy, sensitivity, and specificity of the model including all features increased to 0.875, 0.885, and 0.857, respectively. Additionally, while comparing the AUC values between the SVM models including three morphological features, five spatial features and all eight features, the AUC of the all features model (0.898) was the highest, while that of the spatial feature model (0.886) was higher than the AUC of the morphological feature model (0.862) (Figure 6).

**Table 3:** Classification performance on the test set using SVM.

**Features**	**Acc**	**Sen**	**Spc**	**Yi**	**AUC**
All features	0.875	0.885	0.857	0.742	0.898
Morphological features	0.825	0.826	0.824	0.650	0.862
Spatial features	0.850	0.864	0.833	0.697	0.886

*SVM, support vector machine; Acc, accuracy; Sen, sensitivity; Spc, specificity; Yi, Youden index; AUC, area under the curve*.

## Discussion

In this study, we demonstrated that ultrasound radiomics analysis, based on the spatial and morphological features extracted from ultrasound images, effectively contributed to the preoperative diagnosis of true and pseudo gallbladder polyps, and may be valuable in the clinical management of gallbladder polyps.

For true gallbladder polyps, cholecystectomy is indeed required for the prevention of malignancy development. The 5-year survival rate associated with gallbladder cancer is 2~80%, which is closely correlated to the stage of gallbladder cancer in surgery. The 5-year survival rate of gallbladder carcinoma *in situ* is as high as 80%, while it decreases to 8% in cases with lymph node metastasis, and even drops to values as low as 2% in stage 4b gallbladder cancer (22). Therefore, it is of significance to improve the diagnostic accuracy of gallbladder cancer or precancerous lesions at an early stage.

Recently, several imaging methods have been applied in the examination of gallbladder tumors, such as transabdominal ultrasound, high-frequency ultrasound, contrast-enhanced ultrasound, endoscopic ultrasound, enhanced computed tomography, and enhanced magnetic resonance imaging. As the preferred imaging method for gallbladder lesion examination, conventional trans-abdominal ultrasound is widely used in different levels of hospitals for gallbladder polyp screening and follow-up. However, it is unreliable to distinguish true and pseudo polyps only based on the results of lesion echo, morphology, and blood flow obtained by conventional trans-abdominal ultrasound. Compared to traditional low-frequency ultrasound scans, high-frequency ultrasound scans greatly heighten the accuracy of the determination of the preoperative stage of gallbladder cancer as well as differentiating benign and malignant lesions (23, 24). However, an obvious limitation of high-frequency ultrasound is that it is not effective when the polyps are located deep within the gallbladder body or neck. Moreover, due to the low resolution, contrast-enhanced computed tomography, and enhanced magnetic resonance imaging too do not provide satisfying results in terms of true gallbladder polyp diagnosis.

Owing to the significantly high potential of malignancy development in larger polyps, clinical surgery guidelines highly recommend the performance of cholecystectomy in cases with a gallbladder polyp diameter >1 cm (25). However, this recommendation is being questioned by a growing number of scholars and clinical doctors, with their concerns predominantly centring on the fact that many pseudo non-cancerous gallbladder polyps have a diameter larger than 1 cm and that cholecystectomy performance in such cases may lead to injury and huge wastage of the health system resources. Meanwhile, it has been deemed unreasonable to “watch” the growth of malignant polyps with atypical hyperplasia that have diameters smaller than 1 cm (i.e., 6~10 mm) by ultrasound in the early phase (26–28). Therefore, there is a need for a larger number of studies focusing on the development of new imaging methods to distinguish such true gallbladder polyps for the performance of cholecystectomy as early as possible, as well as efficiently increase the 5-year survival rate of patients and reduce public health resource wastage.

As a medical research hot spot, AI technology is now being applied in medical imaging. In particular, the use of AI in magnetic resonance imaging has proven successful in terms of pathological slide reading (29–32). With the use of computer-based big data analysis, hundreds of unbiased data of image features from existing images can be obtained in a reasonable span of time. With a resolution that far exceeds that of the human eyes, the characteristics for the differentiation of benign and malignant polyps can be obtained by computers from the analysis results of a large number of cases, which can further be used to train computers for deep learning.

In our current study, computer aided high-throughput imaging analysis was applied for the analysis of the medical images of the 99 gallbladder polyps. According to the existing literature, gallbladder cholesterol polyps and adenomas display different patterns of echoes, as obtained by endoscopic ultrasonography (7, 33, 34). Impressively, we found that compared to gallbladder adenomas, cholesterol polyps exhibit a greater degree of unevenness in terms of the pixel distribution of the lesion area and higher aggregability of the highlight area. Meanwhile, our results revealed that the cholesteric polyps exhibited smaller lesion area perimeters and showed greater regularity than the gallbladder tubular adenomas. Particularly, these imaging features of gallbladder cholesterol polyps are closely correlated to their pathophysiological characteristics. Due to the cholesterol crystals in foam cells (3, 6, 35), the images of cholesterol polyps by conventional ultrasound usually show point-like strong echoes or high echoes. For small-size polyps, these echoes are too weak for their detection by the human eye, but may be well-obtained by computers, which have a greater sensitivity. In contrast to the echoes of cholesterol polyps, those of gallbladder adenomas are more uniform in nature as a result of the smaller surface area and similar acoustic impendence inside the adenoma that comprises proliferating glandular epithelial cells and mesenchymal cells. With real-time harmonic contrast ultrasound use, small focal areas of non-enhancement within the peaks could be detected in gallbladder cholesterol polyps, while the enhancements within the peaks usually showed more uniformity in adenomas (8). Strikingly, these newly revealed features are consistent with the pathological characteristics of the lesion. Additionally, these features have potential classification ability. The present study also demonstrated that compared to cholesterol polyps, gallbladder adenomas have a relatively larger girth and volume and show greater shape-related irregularity, consistent with previous reports. Although statistically, the diameter of adenoma is significantly larger than that of cholesterol polyps, for individual cases, we cannot accurately determine true or false polyps by the size of the lesions. In cases with a lesion size of 1 cm with similar echo appearance, it was extremely difficult for the radiologist to provide a pathological diagnosis using conventional ultrasound. However, when combined with AI analysis, including potential morphological and spatial features, a higher diagnostic accuracy in distinguishing true and pseudo gallbladder polyps could be achieved.

Our study also have some limitations. As an initial attempt aimed at the application of up-to-date radiomics technology to distinguishing true and pseudo polyps in the gallbladder, we did not collect a large number of cases. In our following studies, the sample size will be expanded, and deep learning will further be performed on various ultrasound instruments, to provide more promising and reliable parameters for clinical diagnosis. Moreover, we will also attempt to introduce radiomics to the study susing multi-modal ultrasound to obtain more novel indicators. Moreover, combining the automated radiomics technique with the traditional 2D image descriptors, assessed visually by radiologists, could integrate more useful information, which may contribute to more accurate differential diagnosis and deserves further study.

## Conclusion

Ultrasound radiomic analysis based on the spatial and morphology features of original ultrasound images could effectively improve the preoperative diagnostic ability of true and pseudo gallbladder polyps, which may inform gallbladder polyp procedure-related decision-making. Compared to gallbladder adenomas, gallbladder cholesterol polyps showed a greater degree of unevenness and the highlight area showed a higher degree of clustering; these characteristics can be useful in the performance of differential diagnosis in such settings.

## Data Availability Statement

All datasets generated for this study are included in the article/supplementary material.

## Ethics Statement

The studies involving human participants were reviewed and approved by the Institutional Ethics Committee of Fudan University. The patients/participants provided their written informed consent to participate in this study.

## Author Contributions

Each author of the manuscript participated in the study and approved the manuscript for submission. WW and QZ designed the study and guided the data analysis. HY and QY performed the ultrasound scanning and image analysis. YZ and QY performed the data collection and analysis. HY and Q-hY contributed to the manuscript drafting as well as critical revision and editing. All authors approved the final version.

## Conflict of Interest

QZ was a consultant of Hangzhou YITU Healthcare Technology. The remaining authors declare that the research was conducted in the absence of any commercial or financial relationships that could be construed as a potential conflict of interest.

## Abbreviations

GLCM: Gray-level co-occurrence matrix
IMean: Mean of the pixels within the lesion
IMedian: Median of the pixels within the lesion
HE: Histogram entropy
RImedian: Corresponding ratio of the median of the pixels
RImean: Corresponding ratio of the mean of the pixels
Ener: Energy
Cont: Contrast
Entr: Entropy
Homo: Homogeneity
AR: Area ratio
CDD: Center deviation degree
DD: Dispersion degree
Area: Area of the lesion
C.area: Area of minimum convex polygon
Maj.Len: Long axis length of the fitting ellipse
Min.Len: Short axis length of the fitting ellipse
Per: Perimeter
Ori: Orientation
Eq.Dia: Diameter of a circle equal to the lesion area
Sol: Solidity
AUC: Area under the curve
SVM: Support vector machine
AI: Artificial intelligence.
